# Third Trimester Lower Extremity Lymphorrhea

**DOI:** 10.1155/2021/3594923

**Published:** 2021-11-28

**Authors:** Kaori Morimoto, Luke O'Rourke

**Affiliations:** Western University of Health Sciences College of Osteopathic Medicine Pacific Northwest, 200 Mullins Dr., Lebanon OR 97355, USA

## Abstract

**Introduction:**

Lower extremity edema is one of the most common complaints among pregnant patients. However, there is no literature mentioning weeping edema (i.e., lymphorrhea) in a pregnant woman who has no concordant underlying renal and/or cardiac pathology. There is also a lack of evidence and recommendations regarding the therapeutic benefit and safety profile of diuretic use to treat profound pregnancy-associated edema. Herein, we present the case of 32-year-old female who presented with a significant lymphorrhea during the third trimester without cardiac or renal comorbidity and was successfully treated with torsemide. *Case Report*. We report a case of a 32-year-old multigravida patient pregnant with her third child and has two living full-term children (G3P2003). Her pregnancy was complicated by obesity, smoking (vape), and previous history of fetal growth restriction. The patient presented for routine prenatal care at 9-week gestation. She was diagnosed with chronic hypertension at 19 weeks of pregnancy based upon systolic blood pressure > 140. Lifestyle modifications were recommended, but the patient did not comply. At her 31-week office visit, the patient presented with anasarca and clear, slightly viscous fluid seeping through the atraumatic skin of her lower extremities. Preeclampsia, renal, cardiac, vascular, and infectious complications were all ruled out. The patient responded positively to loop diuretic therapy. Torsemide was found to be far more beneficial than furosemide. The patient was induced at 37 weeks secondary to chronic hypertension requiring antihypertensive therapy. Delivery was uncomplicated. The patient gave birth to a healthy male with birth weight of 2,920 g via spontaneous vaginal delivery. *Discussion*. Pitting edema of lower limbs frequently occurs as a result of fluid overload and chronic venous insufficiency, and pregnancy is one of the known risk factors. Additionally, the blockage of lymphatic channel with the gravida uterus likely was the main contributing factor for her lymphorrhea. In this patient, the capillary hydrostatic pressure was likely accentuated due to hypertension, obesity, and vaping. Furosemide was minimally effective to alleviate her symptoms. Torsemide provided much more effective diuresis and symptom control. However, her symptoms persisted until delivery.

**Conclusion:**

Torsemide provided significant therapeutic benefit over furosemide in this patient without adverse maternal, fetal, or neonatal outcomes. Further study is needed to assess the safe use of loop diuretics in the pregnant population who suffers from significant lower extremity edema.

## 1. Introduction

Edema is one of the most common complaints during pregnancy due to increased hydrostatic pressure [1]. This can also contribute to the development of varicose veins in the lower extremity [2]. As the gravid uterus grows, the superficial lymphatic channels in the pelvic area can be obstructed [3]. In this patient, both factors along with chronic hypertension and regular vaping could have contributed to the manifestation of lymphorrhea in her lower extremity. The mechanism is discussed in detail in Discussion.

After ruling out the cardiac and renal causes, the patient was placed on furosemide based on the cardiologist's recommendation. In general, loop diuretics is used with caution during pregnancy as it is known to cross the placenta, and their effect on fetal development is largely unknown [4]. In this patient, torsemide was more effective than furosemide in symptomatic relief. There is no available literature comparing the efficacy of furosemide versus torsemide for the treatment of symptomatic edema and specifically in pregnant patients.

## 2. Case Report

The patient was a 32-year-old Caucasian female pregnant with her third child and had two living full-term children. She was initially seen in the office at 9 weeks and 3 days of gestation. Her initial medications included prenatal vitamin daily, docusate PRN, polyethylene glycol PRN, ondansetron PRN, and topical nystatin PRN. The patient's past medical history included obesity, irritable bowel syndrome, anxiety, nicotine abuse (vape), umbilical hernia, recurrent urinary tract infections, and nephrolithiasis. She had no prior history of hypertension or preeclampsia. Pertinent past pregnancy history included severe fetal growth restriction (FGR) and close interval pregnancy.

Initial prenatal laboratory panel results were benign. The patient's blood type was B positive. She passed the 1-hour oral glucose test at 28 weeks of gestation.

At 19 weeks of gestation, the patient was diagnosed with chronic hypertension secondary to elevated systolic blood pressure > 140. She was started on aspirin 81 mg daily for primary prevention of preeclampsia. She admitted to noncompliance with this recommendation. Her blood pressure remained around 120 s-140 s/70 s-80 s without medication until 31-week gestation.

**Figure 1:**
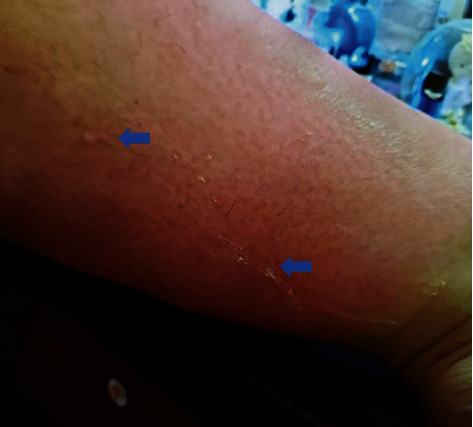
A photograph of the left calf taken by the patient at home. The blue arrows indicate the fluid seeping from the edema. The white residues are dried fluid.

**Figure 2:**
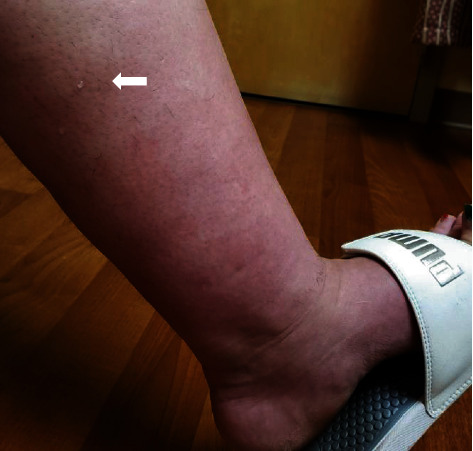
A photograph of the left calf taken by Kaori Morimoto during the prenatal visit. The patient was 31-week and 4-day gestation. Significant bilateral leg edema was noted along with the constant seeping indicated with the white arrow.

Per patient report, her symptoms of the pedal and leg edema, extreme sensitivity to touch, and erythematous discoloration of the caudal half of the shins, consistent with stasis dermatitis, started around 30 weeks of gestation. Over the following days, the patient started noticing a small amount of fluid continuously seeping through the skin of her lower extremities. This rapidly progressed to the point that fluid was streaming down her legs, leaving a trail behind her. The fluid had the appearance of egg white (Figure 1, blue arrows) or water (Figure 2, white arrow). Figure 1 is obtained at home by the patient, and Figure 2 is obtained by the authors during the patient's office visit at 31-week and 4-day gestation with patient permission. As the fluid dehydrated on her skin, its viscosity increased and eventually left the white residue, as seen in Figure 1.

During her office visit at 31 weeks and 4 days of gestation, her blood pressure was 140/100. There was no warmth or tenderness on the affected area during the physical examination. She was treated with oral antibiotics, cephalexin, in case of underlying infection. Unfortunately, antibiotics were of no value in her symptomatic improvement. Over several weeks, it became clear that she did not have cellulitis. Leg elevation and wearing medical compression stockings were not helpful. The patient demonstrated no other classical symptoms of heart failure, nephrotic syndrome, liver failure, or DVT. Urine protein dip was negative, and preeclampsia was ruled out via normal 24-hour urine protein collection. However, due to significant discomfort and profound edema, the patient was admitted to Labor and Delivery for observation, as suggested by the Maternal-Fetal Medicine consultation.

Upon admission to the hospital, the patient received furosemide 20 mg orally per day for symptomatic relief. She had minimal diuresis with this intervention.

While admitted, urinalysis, comprehensive metabolic panel, and complete blood count results were all unremarkable. Well's score was zero. Brain natriuretic peptide level was 33. Fetal heart tones remained category one throughout her stay. Labetalol 200 mg orally twice a day was started for control of chronic hypertension. The patient remained afebrile and normotensive overnight and was discharged on the next day. Outpatient echocardiography showed normal cardiac anatomy and function.

The following week, at 32 weeks and 4 days of gestation, the patient was admitted again for the worsening weeping edema. Chest X-ray and bilateral lower extremity venous duplex ultrasound showed benign findings. However, mild bilateral inguinal lymphedema secondary was noted in the ultrasound report. Upon recommendation by Cardiology, the patient received torsemide 20 mg orally, and she responded with a marked increase in diuresis. Torsemide was remarkably more effective than furosemide for diuresis, edema reduction, and symptomatic relief for this patient. Upon discharge, the patient was started on torsemide 20 mg orally every other day, which she continued for the remainder of her pregnancy.

Throughout the remaining pregnancy, the patient had electrolyte monitoring, and she never required potassium supplementation. Additionally, twice weekly fetal nonstress tests, a weekly ultrasound to follow amniotic fluid index (AFI), and 4-week interval fetal growth ultrasounds were performed to monitor maternal and fetal health closely.

Fetal growth trajectory and amniotic fluid index were unaffected by diuretic use. All fetal nonstress tests were category I and reactive.

The patient reported continuous weeping edema until her delivery at 37-week gestation, but her symptoms were adequately managed so that she could adequately perform activities of daily living (ADLs) without profound discomfort, including caring for her other children.

The patient was induced by Pitocin administration for the indication of chronic hypertension on antihypertensive therapy at 37-week gestation. She successfully delivered a healthy 2,920 g male via spontaneous vaginal delivery after 11 hours of labor. Delivery and postpartum course were uncomplicated.

At the six-week postpartum appointment, her edema was resolved entirely. This suggests that the mechanical obstruction secondary to pregnancy was the likely primary cause of the patient presentation.

## 3. Discussion

Edema is one of the most common complaints during pregnancy. Approximately 70% of women present with clinical edema at some point during pregnancy. One of the common causes of lower extremity edema during pregnancy is an increase in hydrostatic pressure. As a normal physiologic change in pregnancy, total body water increases by 6 to 8 liters. Two-thirds of this fluid is extracellular, and one-third is stored interstitially [5].

Additionally, pregnancy is a risk factor for the development of venous insufficiency and varicosity in the lower extremity and iliac vessels. Varicose veins develop secondary to weakened blood vessel walls and incompetent valves. As the wall stretches, small balloons are formed and contribute to the stasis. While lower extremity vessels are the most vulnerable by gravity, vulvar, rectal, and internal iliac vessels can be affected due to the hemodynamic changes during pregnancy [2].

In its nonpregnant state, the uterus receives about 100 mL/min of blood, less than 3% of the total blood volume. On the other hand, the blood flow to the term uterus approaches 700 mL/min, about 16% of the cardiac output [6]. This increases the amount of blood flowing to the lower half of the body during the pregnancy. Additionally, the dynamic vascular remodeling of uterine vessels and cardiovascular changes accentuates the burden on the venous system.

The lower abdominal and superficial lower extremity lymphatics drain into the superficial inguinal nodes, followed by external iliac nodes, and eventually into the common iliac nodes [7]. Obstructions at any point of this channel can lead to significant lymphedema in the lower extremities and the abdomen. Because of this, lymphatic congestion can be seen during pregnancy secondary to direct compression of the lower extremity lymphatic system by the gravid uterus.

There is limited literature about lymphedema during pregnancy. However, two reported cases of reversible lower abdominal lymphedema in the third trimester showed tortuous dilatation of lymphatic channels, and biopsy confirmed that the obstruction of the lymphatic channels caused those dilatations [3]. Another reported case showed significant bilateral third-trimester leg lymphedema treated with kinesiotherapy [8]. Those symptoms were observed in our patient towards the end of her pregnancy. Additionally, our patient presented with fluid seeping through edematous tissues, which has never been reported previously in any literature.

The risk of vaping during pregnancy in comparison with smoking for humans is largely unknown. One study reported that smoking alters lipid homeostasis while vaping does not in a mouse model [9]. However, many animal studies report the direct toxicity of nicotine in the fetus' developing immune system, neural development, lung function, and cardiac function regardless of the delivery method [10]. Due to the lack of clear warning and addictive potential of nicotine, approximately 7% of women report vaping during pregnancy [11]. Smoking and vaping (e-cigarette use) are both associated with increased risk of hypertension in general. The risk of developing hypertension in people who smoke tobacco or vape regularly compared to those who do not is approximately 1.3 times higher. Furthermore, the risk increases with a combined use of both products to 1.8 times [12]. Therefore, chronic vaping in this patient was likely a contributing factor in developing chronic hypertension during this pregnancy.

Loop diuretics are not the first-line treatment of hypertension in pregnant women and need to be used with caution [4]. Furosemide is known to cross the placenta, and animal reproduction studies have shown adverse events in rat fetuses, including the perinatal growth restriction and subsequent decrease in kidney functions measured by creatinine and uric acid clearance rate [4, 13]. Additionally, the old study conducted by Sibai et al. suggests that a failure in plasma volume expansion at 20- to 25-week gestation may have a correlation with intrauterine fetal demise and intrauterine growth retardation. As shown in animal studies, furosemide decreases plasma volume rather quickly [14, 15]. For those reasons, furosemide was classified as a pregnancy category C drug in the old FDA categorization [4].

ACOG practice bulletin No. 203 lists antihypertensive medications safe during pregnancy. For oral agents, labetalol and nifedipine are most commonly used, followed by methyldopa and hydrochlorothiazide [16]. For chronic hypertension, daily low dose (81 mg) aspirin is recommended (level A), starting between 12 and 28 weeks of gestation [16].

In this patient, torsemide was far more effective than furosemide in edema reduction. The differences between furosemide and torsemide are summarized in the following three points. First, the half-life of torsemide is approximately 3.5 hours, while that of furosemide is 2 hours. Secondly, the bioavailability of oral torsemide is 80%, while that of furosemide is 47%. Lastly, torsemide is 80% metabolized via CYP2C9 in the liver, while furosemide is renally excreted. The onset of diuresis for both medications is within 1 hour [4, 17]. There is no available literature comparing the efficacy of furosemide versus torsemide for treating symptomatic edema in general, nor in pregnant patients.

Because of the lack of study and literature for pregnancy-related lymphorrhea and diuretic use, our case study provides valuable information for managing this rare condition.

## 4. Conclusion

Lower extremity edema is highly prevalent among pregnant women and can cause significant difficulties with ADLs. There is a lack of evidence and safety data regarding the use of diuretic therapy to treat the lower extremity edema during pregnancy. In this case, the patient had profound edema with fluid constantly seeping through the skin without any additional organ dysfunction. Her ADLs were significantly compromised by edema, which was successfully managed with loop diuretics. Torsemide was found to have a significant therapeutic benefit over furosemide in this patient. There were no adverse maternal, fetal, or neonatal effects demonstrated with loop diuretic use in the third trimester. Further study is needed to assess the safe use of loop diuretics in the pregnant population for the symptomatic relief of significant lower extremity edema.

## Data Availability

The data is saved as an electronic medical record in the patient chart of Oregon Medical Group in Eugene Oregon. The image data used to support the findings of this study are included within the article.
